# Clustering of homicide with other adverse health outcomes in the Netherlands

**DOI:** 10.1016/j.pmedr.2022.101988

**Published:** 2022-09-19

**Authors:** J.A. van Breen, M.C.A. Liem

**Affiliations:** Institute of Security and Global Affairs, Leiden University, the Netherlands

**Keywords:** Homicide, Social Disorganization, Suicide, Mortality, Cause of death, Substance abuse

## Abstract

In this project we examine whether homicide ‘clusters together’ with other adverse health outcomes in the Netherlands, focusing on child mortality, suicide, sexual risk behavior, and substance abuse. We expect moderate-to-strong correlations between homicide and the other adverse health phenomena (**hypothesis 1**). Further, we expect that these correlations will be reduced when social disorganization is controlled for (**hypothesis 2**).

The study used population-level data between the years 1996 and 2019, for each of the 40 local regions of the Netherlands. We applied a multilevel correlation procedure to evaluate correlations between homicide and the other adverse health outcomes. Correlations between homicide and the other adverse health outcomes were modest. That is, we found only limited evidence for clustering between homicide and the other adverse health outcomes. The patterns of clustering that did occur, suggested that social disorganization in the region promotes risk-taking behaviors in the population, which ultimately increases rates of homicide, abuse of illegal drugs and births to adolescent parents.

Project materials, syntax and supplementary information can be found on the Open Science Framework at https://osf.io/jd5yu/.

## Highlights

• We examine clustering of homicide with other adverse phenomena in the Netherlands.
• We use population-level data for the 40 regional units, between the years 1996–2019.
• Homicide clusters with births to adolescent parents and substance abuse.
• When levels of social disorganization are accounted for, these clusters disappear.
• There is no evidence that homicide clusters with child mortality or suicide.

## 1 Introduction

Homicides attract considerable public attention, not only as a personal tragedy for those involved, but also as a salient indicator of wider social problems. In the 1830s, early statisticians such as Guerry (Guerry, 1833) and Quetelet (Quetelet, 1835) drew links between homicide rates and adverse social conditions (Oberwittler, 2019). Indeed, it has long been observed that adverse social conditions “cluster together”, often affecting the same communities (Wilson, 1987). However, consideration of homicide as part of these clusters has waxed and waned over time. While early work considered homicide explicitly, it dropped out of view in later work (Liem, 2022). In recent years, homicide has again played a greater role in public health research. For instance, epidemiological approaches study homicide and violence as public health issues that spread through communities in much the same way as infectious disease (Abt, 2019, Slutkin et al., 2015). Here, we build on these approaches, and aim to place homicide in a broader societal context, outside the realm of crime (Hirschi and Gottfredson, 1988), by examining whether homicide co-occurs with other adverse health outcomes in the Netherlands.

Homicide is an adverse health outcome in the sense that it is a cause of death - it is among the 10 most frequent causes of death (Roth et al., 2018) among young people worldwide. Importantly, homicide co-occurs with other adverse phenomena, in the social domain and in the domain of health. These relationships come across not only at the individual level of analysis but also at the *meso*- (e.g. neighborhoods) and macro-level (e.g. countries). In the United States and Canada, homicide rates co-vary with divorce rates, as well as with consumption of drugs and alcohol (Nielsen and Martinez, 2005, Thompson et al., 2001). Similarly, in Europe, national levels of alcohol consumption and unemployment (amongst men in particular) are predictors of a nation’s homicide rate (Ritter and Stompe, 2013, Kivivuori and Lehti, 2012). These relationships are the result of direct links, whereby the occurrence of one phenomenon directly contributes to risk of the other.

We might also consider adverse health outcomes that do not necessarily have direct causal relationships with rates of homicide, but rather co-occur because they have shared causes. For instance, there is a large literature on the relationship between homicide and suicide (Unnithan et al., 1994, Henry and Short, 1954), which suggests that homicide and suicide are different expressions of the same underlying ‘reservoir’ of violence (Unnithan et al., 1994). Likewise, previous research by Pickett and colleagues (Pickett et al., 2005) finds strong correlations at the national-level of analysis between homicide and births to adolescent parents, which is used as an indicator of sexual risk behavior. Ousey (Ousey, 2017) demonstrates that, in the U.S., homicide clusters with sexually transmitted disease, as well as infant mortality. Beyond research focusing specifically on homicide, crime in general has also been connected to adverse health outcomes, such as in self-control theory, which argues that behaviors like drug taking, smoking, and risky sexual behavior are analogous to criminal behavior because they share an element of risk (Hirschi and Gottfredson, 1988).

Taken together, then, previous research shows that high rates of homicide co-occur with higher rates of other adverse health outcomes, including suicide, and alcohol and drug use, but also sexual risk behavior, and infant mortality. In terms of the mechanisms responsible for this clustering, many prominent theoretical frameworks highlight the role of social disorganization (Kubrin and Weitzer, 2003) and social isolation (Burt et al., 2012, Sampson and Wilson, 1995). For instance, Ousey (Ousey, 2017) shows substantial correlations between homicide, infant mortality and sexually transmitted diseases across 524 counties of the U.S., but in models controlling for social disorganization those correlations disappear.

**Fig. 1:**
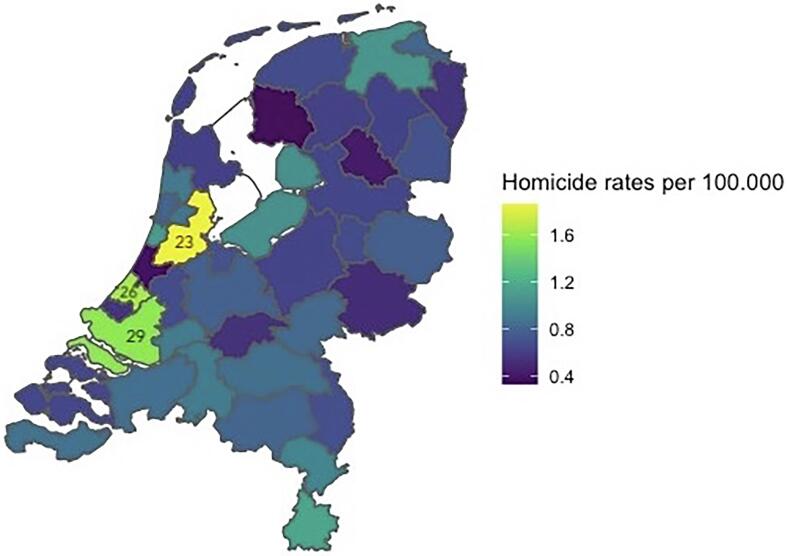
Homicide rates in the 40 regions of the Netherlands, averaged over 1996–2019. The regions with the highest homicide rates are marked with their numbers (23; 26; 29) - these correspond to the metropolitan areas of Amsterdam, The Hague and Rotterdam respectively.

Until now, much of the research on this topic has been conducted in the U.S., which represents a relatively high-disorganization and high-homicide context (Richardson and Hemenway, 2011, National Center for Health Statistics, 2015). Here, we examine if and how clustering between homicide and other adverse health outcomes occurs in the Netherlands. The Netherlands can provide an important test case for the clustering approach because - like much of Western Europe - it is a relatively low-disorganization and low-homicide context. Homicide in the Netherlands ranges from rates of 1.12 per 100.000 to 0.45 per 100.000 in the period under study here (1996–2019). Homicide trends at the regional level are shown in Fig. 1. Levels of social disorganization, too, are relatively low (Eurostat, 2017), the Netherlands has extensive social security programmes (Becker, 2000, Ruijer and Arsenijevic, 2020). As such, the Dutch context differs from the U.S. on several dimensions that are key when applying the clustering approach to homicide. This study will also have implications for our understanding of homicide in Western Europe specifically. Given that rates of homicide in Western Europe are quite low, it is difficult to interpret rates of homicide *per se*. The current approach instead studies the relationship between different phenomena – in this way even low absolute homicide rates can generate patterns of correlations that offer relevant insight into underlying dynamics. Finally, by showing that empirical findings in criminology and public health may implicate similar factors, we stand a better chance of creating effective social policies, and thereby contribute to a healthier and safer society.

### 1.1 The current study

The current study examines clustering of homicide with child mortality, suicide, sexual risk behavior, and substance abuse, in the Netherlands between 1996 and 2019. Table 1 shows the basic rates of the adverse health outcomes during the period under study. Following previous research (Thompson et al., 2001, Ousey, 2017), we expect moderate-to-strong correlations amongst the phenomena (**hypothesis 1**), which are reduced in size when controlling for indicators of social disorganization (**hypothesis 2**).

**Table 1:** Incidence rates per 100.000 of the population for Homicide, Child Mortality, Births to adolescent parents, Suicide, and Substance abuse in the Netherlands between 1996 and 2019.

	**Average rate** over the period	**Max. rate** (associated year)	**Min. rate** (associated year)
Homicide deaths	0.81	1.12 (2004)	0.45 (2016)
Child mortality	0.97	1.38 (1996)	0.74 (2018)
Births to adolescent parents	3.18	4.66 (2003)	1.08 (2019)
Suicide deaths	10.21	11.96 (2017)	8.60 (2008)
Legal substance abuse	43.04	59.28 (2007)	34.63 (2000)
Semi-legal substance abuse	15.69	26.78 (2010)	6.94 (1998)
Illegal substance abuse	15.95	22.45 (1996)	12.07 (2012)

Note. Data sources: Dutch Homicide Monitor; *CBS,* 2021; *LADIS*, 2021.

## 2 Method

### 2.1 Study design

The study applies a correlational design to population-level data. From national-level databases we extract data on the different adverse health outcomes in each of the 40 regions of the Netherlands, in each year. The regional units (“COROP-regions”) correspond to NUTS3 in the EU-register, and represent the intermediate level between council areas and provinces. The regions are specified as a central nucleus (normally a city) and its environs. Fig. 1 gives a visual overview of the 40 regions. We chose this regional scale to maximize the variance in the central indicators. A small unit may go many years without any homicide incidents, larger units of analysis minimize this issue. Additionally, the focus on regional units of analysis is in line with previous research studying homicide clustering (Ousey, 2017).

### 2.2 Data sources

We gathered population-level data from three data sources. Homicide data was taken from the Dutch Homicide Monitor (Ganpat et al., 2012), which draws on police sources triangulated with archival searches, to identify homicide incidents across the Netherlands from 1992 onwards. We sourced the substance abuse data from the National Alcohol and Drugs Information System (*LADIS*). LADIS compiles data from all drug addiction rehab facilities across the country. The data on child mortality, births to adolescent parents, suicide, demographics, and social disorganization was taken from Statistics Netherlands (*CBS*). Each of these databases allow for a regional and yearly breakdown of the data.

### 2.3 Variables

#### 2.3.1 Central variables

The homicide and suicide variables reflect cases where homicide/suicide is the registered cause of death, expressed per 100.000 of the population. Child mortality was operationalized as deaths of children under 5 years of age (excluding those due to homicide) expressed per 100.000 of the population. Sexual risk behavior was operationalized as births to adolescent parents (Pickett et al., 2005) - the number of girls between 13 and 19 years old who give birth, expressed as a percentage of the total number of adolescent girls. Adolescent pregnancies are associated with abusive romantic relationships and coercion (Klein, 2005), as well as depression, and low birth weight in the child (Picavet et al., 2014). As such, it is considered an adverse outcome in many countries, including the Netherlands (Picavet et al., 2014). The substance abuse variable reflects the number of people entering rehabilitation services for substance abuse, again expressed per 100.000 of the population. Individuals admitted more than once within a year are counted only once. We create three indicators of substance abuse, based on the legality of the substance: Legal (alcohol), Semi-legal (cannabis, medicines)^1^ and Illegal (opiates, cocaine, amphetamines, ecstasy, and GHB).

#### 2.3.2 Explanatory mechanisms

We consider regional demographics, and social disorganization as explanatory mechanisms for the correlations amongst the phenomena.

##### 2.3.2.1 Regional demographics

To capture the demographic features of the regions, we consider the age of the population, population density in the region, and population growth. Age of the population was operationalized as i) percent of the population under 25 years of age, and ii) percent of the population over 65 years of age. Population density (iii) was operationalized as the number of inhabitants per km^2^. Population growth (iv) in the region was calculated as the size of the region’s population relative to the previous year.

##### 2.3.2.2 Social disorganization

Following Ousey (Ousey, 2017) we assess the contribution of social disorganization, through five indicators reflecting poverty, social (in)stability and population heterogeneity. As an indicator of poverty, we use Gross regional Product of the region per head of the population. As indicators of social (in)stability, we include the percentage of single parent households, and residential mobility, which have been established by previous research as indicators of deprivation in Europe (Bailey and Livingston, 2008, Chzhen and Bradshaw, 2012). As indicators of population heterogeneity, we include i) the percentage of the region’s population who were born abroad, and ii) the percentage of the region’s population with a ‘Non-Western migration background’. This categorization is taken from *Statistics Netherlands,* and includes first- and second-generation immigrants from “Non-Western” countries of origin.^2^ Previous research has established a link between areas with high populations of migrants and social disorganization in Europe (Berti et al., 20142014, Sociaal Cultureel Planbureau, 2019).

### 2.4 Ethical considerations

Our analysis relies on population-level data from which no individual scores can be discerned. This project was approved by the faculty ethics board.

### 2.5 Analytical procedure

We first examine spatial and temporal interdependencies. Temporal interdependence occurs when (e.g.) homicide rates in a given year predict homicide rates in the following year. Spatial interdependence occurs when (e.g.) homicide rates in a given region are affected by homicide rates in the adjoining regions. We calculate a spatial lag term – for each region we averaged the homicide rates in the adjoining regions (Ousey, 2017). Evidence for temporal interdependence was examined with a hierarchical linear regression model, in which the Year the observations were taken from was entered as a level 2 factor with an auto-regressive structure (AR1). Indeed, a model including the higher-order term Year was a significantly better fit to the data than a model without the higher-order term, χ^2^ (1) = 27.737, *p* <.001. However, there was no evidence for spatial interdependencies, all *t-*values < 1.70, all *p*-values > 0.243. The models reported below control for the temporal interdependency between the observations. The models reported below control for the temporal interdependency between the observations.

The analysis is composed of three steps. The first step evaluates support for hypothesis 1 – that homicide correlates with the other adverse health outcomes. We use the multilevel correlation procedure in R (R Core Team. R, 2020, Makowski et al., 2020), with Year (1996–2019) as the superordinate factor to account for the temporal interdependence. The second and third steps of the analysis examine the explanatory mechanisms that drive the correlations observed in the first step. Step 2 extends the basic model with the inclusion of the regional demographics. In step 3 we add the social disorganization indicators, to examine evidence for hypothesis 2, which suggests that the correlations between the adverse health phenomena will be reduced when the influence of social disorganization is partialled out.

In evaluating the strength of a correlation we follow Cohen’s (Cohen, 1988) guidelines, considering correlations above 0.30 as moderate, and correlations above 0.50 as strong.

## 3 Results

### 3.1 Clustering

We hypothesized that homicide would show moderate-to-strong correlations with the child mortality, suicide, births to adolescent parents, and substance abuse (legal, semi-legal and illegal). As shown in Table 2, correlations were low-to-medium, ranging from *r* = 0.009 to *r* = 0.32. The largest correlations were observed between homicide and abuse of illegal substances, *r* = 0.32, and between homicide and births to adolescent parents, *r* = 0.22. That is, high rates of homicide tended to co-occur with higher rates of illegal substance abuse, and more frequent births to adolescent parents. The relationship between homicide and abuse of legal and semi-legal substances also reached significance, but the correlation coefficients were lower (*r* = 0.14 and *r* = 0.11 respectively) than in the case of illegal substances. The correlations between homicide and suicide, and homicide and child mortality did not reach significance. Overall, then, support for hypothesis 1 was modest.

**Table 2:** Correlations between homicide and the other indicators, before the inclusion of the explanatory mechanisms. The model controls for Year at Level 2.

	**Correlation of homicide rate with other adverse outcomes**		
	Correlation coefficient	95 % CI [lower bound, upper bound]	p-value
Child mortality	*r* = 0.009	[−0.05, 0.07]	*p*=.758
**Births to adolescent parents**	***r* = 0.22**	[0.16, 0.29]	***p*<.001**
Suicide	*r* = 0.04	[−0.02, 0.10]	*p*=.229
**Alcohol abuse**	***r* = 0.14**	[0.07, 0.21]	***p*<.001**
**Abuse of semi-legal substances**	***r* = 0.11**	[0.05, 0.18]	***p*=.001**
**Abuse of illegal substances**	***r* = 0.32**	[0.26, 0.38]	***p*<.001**

### 3.2 Explanatory mechanisms

The second step of the analysis incorporates regional demographics that may influence the clustering of homicide with the other adverse phenomena. The full models are shown in Table 3 (left columns). Including these variables did not impact the relationship between homicide and the percentage of births to adolescent parents – the partial correlation coefficient remained the same as it was before (*r* = 0.22). The partial correlation between homicide and abuse of illegal substances remained significant at *r* = 0.22, although it was somewhat reduced from its previous value of *r* = 0.32. Similarly, the partial correlations between homicide and i) legal and ii) semi-legal substance abuse were also reduced by the inclusion of the demographic variables The partial correlation coefficient between homicide and legal substance abuse dropped from *r* = 0.14 to *r* = 0.05. The partial correlation coefficient between homicide and semi-legal substance abuse dropped from *r* = 0.11 to *r* = 0.07.

**Table 3:** Correlations between homicide and the other indicators, with explanatory mechanisms included. Both models shown here control for Year at Level 2.

	**Correlations of homicide rate with other adverse outcomes**					
	**Model including demographics**			**Model including demographics + disorganization indicators**		
	Correlation coefficient	95 % CI [lower, upper]	p-value	Correlation coefficient	95 % CI [lower, upper]	p-value
Child mortality	*r =* 0.05	[-0.02, 0.11]	*p =* .555	*r =* 0.02	[−0.05; 0.09]	*p>*.999
**Births to adolescent parents**	***r =* 0.22**	**[0.16; 0.29]**	***p*<.001**	*r* = 0.06	[−0.01; 0.13]	*p*>.900
Suicide	*r =* 0.05	[−0.01; 0.12]	*p =.*193	*r* = 0.05	[−0.02; 0.12]	*p*>.999
Alcohol abuse	*r* = 0.05	[−0.02; 0.12]	*p* =.393	*r* = 0.00	[−0.08; 0.08]	*p*>.999
Abuse of semi-legal substances	*r* = 0.07	[ 0.00; 0.14]	*p* =.223	*r* = −0.02	[−0.09; 0.06]	*p*>.999
**Abuse of illegal substances**	***r* = 0.22**	**[0.15; 0.28]**	***p*<.001**	*r* = 0.06	[−0.02, 0.14]	*p*>.900

At this stage of the analysis two significant correlations remain: between homicide and births to adolescent parents, and between homicide and abuse of illegal substances. We examine whether indicators of social disorganization can explain these correlations (right columns of Table 3). The inclusion of these indicators reduced the partial correlations between homicide and births to adolescent parents from *r* = 0.22 to non-significance, *r* = 0.06, *p* >.900. Similarly, the partial correlation between homicide and the abuse of illegal substances dropped from r = 0.22 to non-significance, *r* = 0.06, *p* >.900. Thus, there was support for hypothesis 2 - although the clustering between homicide and the other phenomena was modest to begin with, the clustering that was observed is attributable to social disorganization.

The Appendix describes some supplementary analyses: a brief discussion of predictive relationships between social disorganization and each of the adverse health outcomes, and a model including an additional demographic variable.

## 4 Discussion

Overall, evidence for clustering between homicide and the other adverse health outcomes was limited - there was only modest support for hypothesis 1. It seems that in the Netherlands, low rates of homicide within a given region do not necessarily mean that other adverse health outcomes are also infrequent. Hypothesis 2 stipulated that the correlations between homicide and the other adverse health outcomes would be reduced when social disorganization is included in the model. Indeed, there was support for hypothesis 2 – suggesting that levels of social disorganization were responsible for the small degree of clustering we did observe. Contrasting these findings with those from the United States (Ousey, 2017), it seems that, in the Netherlands, the clustering of adverse health outcomes is less strong than in the U.S. However, as in the U.S., social disorganization appears to be a key driver of any clustering that does occur.

Although there was limited evidence for ‘general’ clustering, there were some more nuanced patterns that are worth discussing. As we have seen, rates of homicide were associated with births to adolescent parents, and substance abuse (especially illegal substances), but not with the other *lethal* outcomes - child mortality or suicide. This pattern begs the question of what differentiates suicide and child mortality, from births to adolescent parents and substance abuse. Previous research often considers drug-taking, births to adolescent parents, and crime to be outcomes of risk-taking behaviour (Hirschi and Gottfredson, 1988, Pickett et al., 2005, van Nieuwenhuijzen et al., 2009). Births to adolescent parents and substance abuse may start as general risk behaviors by the individual (recreational drug use and unprotected sex) which escalate to the more serious outcome (addiction and unplanned pregnancy). Child mortality or suicide on the other hand, are less clearly the result of risk behaviors. For instance, the classification of a death as suicide requires establishing some form of intent (Brookman, 2015, Lindqvist and Gustafsson, 2002, Supreme Court, 2020) – any death resulting from risky behavior is more likely to be classed as accidental. Individual risk behavior has also been linked to social disorganization. Risk-sensitivity theory suggests that individuals who experience social disorganization are not able to meet their needs through conventional low-risk means, and therefore switch to high-risk strategies (Mishra and Novakowski, 2016). In other words, social disorganization produces risk-taking behavior in individuals. Recent research from Europe has also generated evidence for the opposite causal direction (Airaksinen et al., 2021, Sariaslan et al., 20132013). In this case, individuals with tendencies towards risk behavior (self-)select into certain areas, which then contributes to disorganization in the area. In sum, then, there are several mechanisms by which individual risk behavior might interact with social disorganization to produce clusters of adverse outcomes such as homicide, substance abuse, and births to adolescent parents (but not suicide or child mortality).

However, in the current study we do not focus on the individual level of analysis, but rather on *regions.* As such, our findings suggest that certain *regional populations* are characterized by risk behaviors, (in part) under the influence of social disorganization in the region This approach fits with our finding that homicide shows a medium correlation with illegal substance abuse, but correlations with legal and semi-legal substance abuse are lower. Illegality is clearly linked to risk, which suggests that the illegal aspect of illegal substance abuse contributes to its correlation with homicide. Though we must be cautious in applying individual-level theories to regional-level findings, we believe it is interesting to consider risk behavior in the regional population as a possible explanation for the precise patterns of clustering we observe between homicide and substance abuse, and homicide and births to adolescents, but not homicide and child mortality or suicide.

In terms of the limitations associated with this work, the indicators we use are not pure reflections of the concepts under study. For instance, our measure of substance abuse relies on those *seeking treatment* for substance abuse. Reflecting on this limitation, we believe the results of the analysis provide a degree of reassurance on the functioning of the substance abuse variables. Homicide and the substance abuse variables are positively correlated, a finding which would be expected if the substance abuse variables are an adequate proxy for (problematic) drug use. If they instead reflected the sensible decision to seek treatment, their positive correlation with homicide would be counter-intuitive. Nevertheless, future work should explore alternative ways of capturing problematic drug use.

One additional limitation concerns the level of analysis, which relied on regional units. Current trends in criminological research prefer smaller units of analysis, but low rates of homicide prevented us from doing so - many small units will not experience any homicides in a given year, leading to empty cells in the homicide variable. Future work might fruitfully compare different European countries to obtain larger samples of homicides. Our homicide data is drawn from the Dutch Homicide Monitor, which has a European counterpart (the European Homicide Monitor – EHM (Granath et al., 2011, Liem et al., 2013). One benefit of the EHM specifically is that it codes *subtypes* of homicide, which could allow us to examine more specific clustering patterns. For instance, substance abuse may cluster specifically with *drug-related* homicides. One further reflection on the level of analysis is whether we would have observed stronger (or indeed weaker) effects at smaller levels of analysis. Smaller levels of analysis require less aggregation and as such nuanced patterns might come across more strongly. On the other hand, larger levels of analysis tend to generate stronger correlations, so at smaller levels of analysis we might expect less strong effects. The issue of how effects at different levels of analysis relate is a complex one, and this issue represents a key area for future research.

More in-depth analysis of spatial and temporal patterns is another key area for future research. In our view, an analysis of trends over time would be of particular interest. Homicides in the Netherlands have declined over the years under study here, and so has – for instance – child mortality (Centraal Bureau voor Statistiek, 2020). Future work might draw on time-series models, to examine whether trends in homicide rates are related to trends in other phenomena. Such findings would provide further evidence that homicide and other adverse health outcomes respond to similar external influences.

### 4.1 Conclusion

We examined whether homicide clusters together with child mortality, suicide, births to adolescent parents, and substance abuse, in the Netherlands between 1996 and 2019. We found only limited evidence for such clustering. The patterns of clustering that did occur, suggested that social disorganization of the local area promotes risk-taking behaviors in the population, which increases rates of homicide, substance abuse and births to adolescent parents. Ultimately, we hope that this work will offer a new perspective on homicide to academics as well as policymakers. Aside from its links to crime (established in the criminological literature (Berg, 2019, Minkov and Beaver, 2016, Fajnzylber et al., 2000) homicide also has relevant links to adverse health outcomes.  

**Ethical approval:** This project was approved by the faculty ethics board under number 2021–009-ISGA.

### CRediT authorship contribution statement

**J.A. van Breen:** Conceptualization, Methodology, Investigation, Data curation, Writing – original draft, Writing – review & editing. **M.C.A. Liem:** Conceptualization, Writing – review & editing.

## Declaration of Competing Interest

The authors declare that they have no known competing financial interests or personal relationships that could have appeared to influence the work reported in this paper.

## Figures and Tables

**Table A1:** Central findings without (left) and with (right) the Randstad variable included under regional demographics.

	**Correlations of homicide rate with other adverse outcomes**					
	**Model with demographics (excluding Randstad)**			**Model with demographics + Randstad**		
	Correlation coefficient	95 % CI [lower, upper]	p-value	Correlation coefficient	95 % CI [lower, upper]	p-value
Child mortality	*r =* 0.05	[−0.02, 0.11]	*p =*.555	*r = 0.*05	[−0.02; 0.11]	*p >*. 999
Births to adolescent parents	*r =* 0.22	[0.16; 0.29]	*p* <.001	*r* = 0.22	[0.16; 0.29]	*p* <.001
Suicide	*r = 0.*05	[−0.01; 0.12]	*p =.*193	*r* = 0.05	[−0.01; 0.12]	*p* =. 625
Alcohol abuse	*r* = 0.05	[−0.02; 0.12]	*p* =.393	*r* = 0.06	[−0.01; 0.12]	*p* =.717
Abuse of semi-legal substances	*r* = 0.07	[ 0.00; 0.14]	*p* =.223	*r* = 0.07	[0.00; 0.14]	*p* =.389
Abuse of illegal substances	*r* = 0.22	[0.15; 0.28]	*p* <.001	*r* = 0.22	[0.15; 0.28]	*p* <.001

**Table B1:** Does social disorganization predict each of the adverse health outcomes?

	**Explanatory power of predictive model**		
	**Year only**	**Year + Deprivation indicators**	**Improvement in predictive power**
Homicide	7 %	33 %	χ^2^(5) = 106.71, *p* <.001
Births to adolescent parents	30 %	66 %	χ^2^(5) = 207.90, *p* <.001
Illegal substances	8 %	43 %	χ^2^(5) = 140.66, *p* <.001
Semi-legal substances	50 %	51 %	χ^2^(5) = 83.12, *p* <.001
Legal substances (Alcohol)	16 %	24 %	χ^2^(5) = 51.91, *p* <.001
Child Mortality	24 %	29 %	χ^2^(5) = 37.13, *p* <.001
Suicide	14 %	17 %	χ^2^(5) = 68.30, *p* <.001

Note: there are five indicators of disorganization which are added to the model. Their combined influence as “social disorganization” is captured by increases in the model fit – a model including these 5 indicators is better at predicting rates of (e.g.) homicide than a model without these indicators.

## Data Availability

Data will be made available on request.

## Appendix

### Additional regional features

Aside from the regional demographics already included in the models (population density, population growth/shrinkage; age of the population), in this section we consider one other co-variate, namely whether or not a region is part of the “ Randstad”. The Randstad is a large metropolitan area which is essentially a confluence of the four major cities (Amsterdam; Rotterdam; Utrecht; the Hague), and suburban towns connecting them. We created a binary variable reflecting whether the area was part of de Randstad (0 = No; 1 = Yes), based on information published by Statistics Netherlands (Centraal Bureau voor Statistiek, 2018) (Table 2.1). Adding this additional variable did not impact the clustering effects, as shown in Table A1 below. The left columns repeat the findings reported in the manuscript text, the right columns show the revised findings including the Randstad covariate.

### Predictive effects of social disorganization

As described in the manuscript text, the disorganization indicators impact the *relationships between* the adverse health outcomes. Here, we consider whether the disorganization indicators can predict each of the outcomes alone. In these models we again control for Year at level 2. In each case, the model including the disorganization indicators provided significantly better fit to the data than an empty model, as shown in Table B1. That is, social disorganization can predict the adverse health outcomes directly, as well as playing a role in the clustering between these three phenomena.

## Footnotes

1 For cannabis, personal use (including possession and purchase) is legal in the Netherlands, but the market is highly regulated. The production of cannabis, moreover, remains a legal gray area (Korf, 2019). Similarly, medicines were classified as semi-legal, as the substances themselves are often legal, but those who become addicted often turn to illegal or fraudulent means to acquire them.

2 Non-Western countries of origin include all countries in Africa, Latin and South America, and Asia, except Indonesia and Japan.
